# Long-Term Motor Deficit and Diffuse Cortical Atrophy Following Focal Cortical Ischemia in Athymic Rats

**DOI:** 10.3389/fncel.2019.00552

**Published:** 2019-12-17

**Authors:** Charlotte M. Ermine, Fahad Somaa, Ting-Yi Wang, Brett J. Kagan, Clare L. Parish, Lachlan H. Thompson

**Affiliations:** Florey Institute of Neuroscience and Mental Health, Parkville, VIC, Australia

**Keywords:** diaschisis, staircase test, neurodegeneration, infarction, sensorimotor, stroke

## Abstract

Development of new stroke therapies requires animal models that recapitulate the pathophysiological and functional consequences of ischemic brain damage over time-frames relevant to the therapeutic intervention. This is particularly relevant for the rapidly developing area of stem cell therapies, where functional replacement of circuitry will require maturation of transplanted human cells over months. An additional challenge is the establishment of models of ischemia with stable behavioral phenotypes in chronically immune-suppressed animals to allow for long-term survival of human cell grafts. Here we report that microinjection of endothelin-1 into the sensorimotor cortex of athymic rats results in ischemic damage with a sustained deficit in function of the contralateral forepaw that persists for up to 9 months. The histological post-mortem analysis revealed chronic and diffuse atrophy of the ischemic cortical hemisphere that continued to progress over 9 months. Secondary atrophy remote to the primary site of injury and its relationship with long-term cognitive and functional decline is now recognized in human populations. Thus, focal cortical infarction in athymic rats mirrors important pathophysiological and functional features relevant to human stroke, and will be valuable for assessing efficacy of stem cell based therapies.

## Introduction

Pre-clinical development of new therapies for stroke is critically dependent on animal models of ischemic brain damage that produce functional impairments relevant to human stroke outcomes. The stability of these impairments and sensitivity to different behavioral tests can vary widely depending on the nature of the ischemic event, the timing of the testing after damage and the animal species and strain (Carmichael and Chesselet, 2002; Schaar et al., 2010; Trueman et al., 2016). This can have an important baring on the design and interpretation of pre-clinical tests of efficacy for novel therapies. Importantly, functional deficits targeted by new therapies should be stable over a time-course relevant to the therapeutic mechanism and distinguishable from those that resolve spontaneously and independently of any treatment.

This presents particular challenges for stem cell therapies, which aim to restore function in stroke patients through intra-cerebral transplantation of cells that can replace damaged neuronal circuitry. Recent studies using human pluripotent stem cells have shown that grafted neurons require months, rather than weeks, to acquire the mature electrophysiological properties necessary to replace functional neurons (for review see, Thompson and Bjorklund, 2015). Thus transient functional deficits limited to the acute phase after stroke, such as gross motor function assessed by rotarod performance in certain rodent stroke models (Hunter et al., 2000; Zhang et al., 2000; Bouet et al., 2007), are unsuitable as tests of the potential benefit of cell replacement therapies. Furthermore, preclinical work in this area requires the use of chronically immune-compromised animals in order to prevent rejection of xeno-grafted human cells.

Here we sought to establish a model of ischemia in athymic (‘nude’) rats resulting in motor deficits that persist over a time frame that is clinically meaningful for assessment of efficacy of human cell-based restorative therapies. We chose a model of focal cortical ischemia induced through local injection of the vasoconstrictor endothelin-1 (ET-1). Previous studies have shown that cortical injection of ET-1 recapitulates important pathophysiological aspects of human ischemia including significant reduction in cortical blood flow, persisting up to 23 h (Schirrmacher et al., 2016), leading to hypoperfused tissue and development of an infarcted area associated with neuronal cell loss (Windle et al., 2006; Nguemeni et al., 2015; Weishaupt et al., 2016).

Behavioral studies in rodents have reported impairment in both motor (Adkins et al., 2004; Gilmour et al., 2004; Soleman et al., 2010) and cognitive function (Cordova et al., 2014; Deziel et al., 2015; Livingston-Thomas et al., 2015) after microinjection of ET-1 into the sensorimotor cortex. Tests of learning and memory and executive function have shown impairment in certain tasks that persist up to 18 weeks after ischemia (Livingston-Thomas et al., 2015). Motor performance has been investigated more extensively, where a number of studies have shown deficits in a range of motor tasks, however, the follow-up time has typically been limited to 2–4 weeks post-ischemia, although impairment in a forelimb reaching task up to 12 weeks post-ischemia has been reported (Gilmour et al., 2004).

The aim of this study was to assess the stability of motor impairment over a significantly longer timeframe and feasibility of the model in immune compromised rats. We also report results of post-mortem histological analysis showing chronically progressive atrophy of the infarcted cortical hemisphere.

## Materials and Methods

### Animals

All procedures were conducted in accordance with the Australian National Health and Medical Research Council’s published Code of Practice for the Use of Animals in Research, and experiments were approved by the Florey Institute of Neuroscience and Mental Health Animal Ethics Committee.

A total of 58 male athymic (CBH^rnu^) rats at 8 weeks of age were used at the beginning of this study. The animals were group housed in individually ventilated cages with Alpha-dri paper bedding material (Abel Scientific, Perth) to reduce skin and eye irritation and housed on a 12 h light/dark cycle with *ad libitum* access to food and water.

The study design involved the establishment of a group of 44 animals with focal cortical ischemia induced by local injection of ET-1 and a control group of 14 animals injected with saline at the same location. Animals were tested for motor function at 1, 2, 4, 8, 16, 24, and 36 weeks after ET-1/Saline injection. The staircase pellet retrieval test was used as the primary measure and a subset of animals were also tested for gross motor function on the accelerating rotarod. At the completion of the study at 9 months, we also elected to test forepaw function using the adjusted stepping test. A separate cohort was used for histological analysis at each corresponding time-point. Four animals were also taken at 3 days in order to measure infarct volume. All of the saline injected animals were taken for post-mortem histology at 9 months. Long-term experiments with athymic rats present particular challenges with respect to maintaining the health and well-being of the animals.

Spontaneous development of skin irritation and respiratory complications are not unusual, even in certified clean facilities. This lead us to euthanize 19 animals at various time-points beyond 3 weeks and these were excluded from histological and behavioral analysis. The experimental design is presented below.


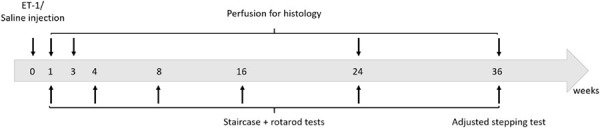


### Endothelin Induced Ischemia

All surgeries were performed under general anesthesia using 3% isoflurane delivered in O_2_. The rats were fixed in a flat skull position in a stereotaxic frame (Kopf, Germany) and 0.5 μl of either 0.9% sterile saline (*n* = 14) or 800 pmol/μl ET-1 (AusPep, Melbourne) in sterile saline (*n* = 44) was delivered at each of two sites in the frontal cortex (a total of 1 μl delivered) using a glass capillary attached to a 5 μl micro-syringe as previously described (Windle et al., 2006). The stereotaxic co-ordinates were: 0.5 and 2 mm rostral to Bregma; 2.8 mm lateral to Bregma (right hemisphere) and 1.5 mm below the dural surface. The solution was delivered at a rate of 0.5 μl/min. There was consistently reflux of some solution up the cannula and the solution was allowed to sit on the surrounding cortical surface.

### Rotarod Test

Gross motor function was assessed on an accelerating rotarod within a 5 min test period. Before testing, a training period was conducted with one steady session at 16 rpm and two ramping sessions at 4–40 rpm over 5 min with 10 min rest intervals in between each. Testing was conducted with two sessions at 4–40 rpm over 5 min with a 10 min rest interval and the average latency to fall recorded (sec) was recorded. Animals were tested at 1 week and 4, 8, 16, 24, and 36 weeks after injection of saline (*n* = 5) or ET-1 (*n* = 7). All tests were performed blinded to saline or ET-1 treatment.

### Staircase Test

**FIGURE 1:**
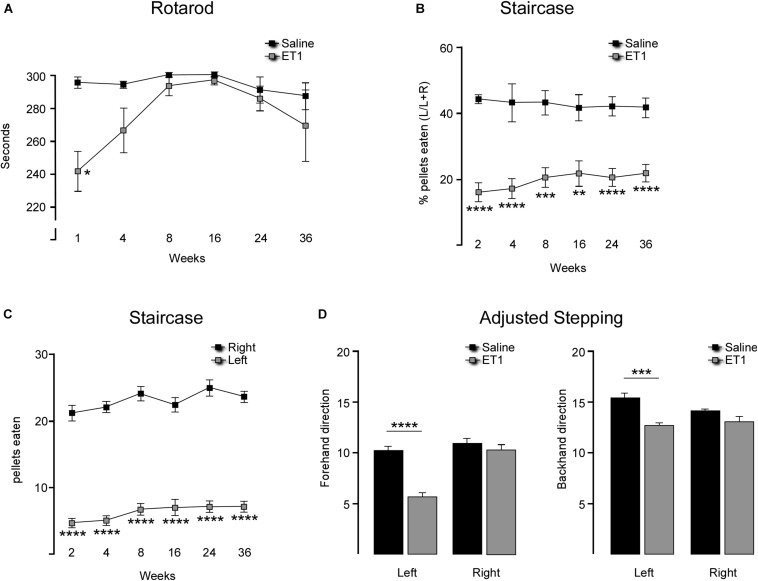
Longitudinal comparison of motor performance in rats injected with saline or ET-1. **(A)** Mean latency to fall (±SEM) on the accelerating rotarod was significantly shorter for ET-1 treated animals (*n* = 7) at 1 week but not significantly different from saline controls (*n* = 6) from 4 weeks and later [two way ANOVA with Sidak’s multiple comparison: Interaction factor *F*_5_,_63_ = 1.817, *p* = 0.111, Time factor *F*_5_,_63_ = 2.536, *p* = 0.0373, Group factor *F*_1_,_63_ = 10.42, *p* = 0.002; *t*(63) = 3.829; ^∗∗^*p* < 0.0018]. **(B)** Successful pellet retrieval using the forelimb contralateral to the injected hemisphere (left forelimb) was significantly lower for ET-1 treated animals (*n* = 18) compared to the saline controls (*n* = 8) at all time-points tests up to 36 weeks (two-way ANOVA with Sidak’s multiple comparison: Interaction factor *F*_5_,_121_ = 0.5086, *p* = 0.7693, Time factor *F*_5_,_121_ = 0.1052, *p* = 0.9909, Group factor *F*_1_,_121_ = 129.6; *p* < 0.0001; ^∗∗^*p* = 0.0012, ^∗∗∗^*p* < 0.001, ^****^p < 0.0001). **(C)** Total pellets retrieved with each forelimb shows a significant reduction in skilled use of the left forelimb in ET-1 animals (*n* = 18) compared to the saline controls (*n* = 8) at all time-points tests up to 36 weeks (two-way ANOVA with Sidak’s multiple comparison: Interaction factor *F*_5_,_194_ = 0.3998, *p* = 0.8485, Time factor *F*_5_,_194_ = 3.171, *p* = 0.0089, Group factor *F*_1_,_194_ = 911.6, *p* < 0.0001; ^****^p < 0.001). **(D)** At the 36 week time-point, ET-1 treated animals (*n* = 7) displayed a significant reduction in their capacity to make adjusted, weight-baring steps with the left forelimb compared saline controls (*n* = 7) in both the backhand and forehand directions (*t*-test for each forelimb; ^∗∗∗^*p* = 0.0002; ^****^p < 0.0001) while there was no difference in performance for the right forelimb between treatment groups. All data shown as the group mean ± SEM.

Skilled forepaw use was assessed using the staircase test originally described by Montoya et al. (1991) and modified by Winkler et al. (1999). Briefly, the animals were placed in a staircase apparatus (Campden Instruments, United Kingdom) in a dark room where each forepaw had unilateral access to sugar pellets (35 mm, Able Scientific, Canning Vale) positioned on an ascending set of steps. Ten pellets were placed on each of steps 2–6 for a total of 50 accessible pellets per forelimb. The total number of pellets consumed was scored for each forelimb over a 20 min test period. All animals were placed on a food-restricted diet such that weight during the test period was 80–90% of the pre-test free-feeding weight. A training period was required to achieve a stable level of performance for the unimpaired forelimb (contralateral to saline/ET-1 injection) so that animals were tested once a day over 7–10 days. Animals that were not able to retrieve a minimum of 20 pellets with the unimpaired forelimb were not included for further testing. The number of pellets consumed was recorded as the average performance over the last 3 days of testing. The first test was initiated 4 days after surgery and completed by 2 weeks post-surgery (represented as ‘2 week’ time-point, Figure 1). Animals were again tested at 4, 8, 16, 24, and 36 weeks – for these later time-points the weeks indicate the initiation of testing. All tests were performed blinded to saline (*n* = 8) or ET-1 treatment (*n* = 18).

### Adjusting Stepping

This test was only included at the final, long-term time-point of 36 weeks post-surgery as an additional measure of motor function. Based on procedures originally described by Schallert et al. (1979) and modified by Olsson et al. (1995) and Winkler et al. (1999), rats were assessed for their ability to make stepping adjustments to a weight-bearing forelimb as it is moved laterally along a smooth surface. Rats were held by the experimenter so that one forelimb was allowed to make weight-bearing contact with the bench and the rats were moved laterally in both directions (forehand and backhand) over a 1 m distance. This was repeated for each forelimb and the number of adjustment steps was recorded. This test required a training period for stable performance by the unimpaired forelimb. Rats were tested twice per day for 7–10 consecutive days and the final performance reported is the average score over the last 2 days of testing (4 sessions). All tests were performed blinded to saline (*n* = 6) or ET-1 treatment (*n* = 7).

### Tissue Processing and Histology

Animals injected with ET-1 were taken for histological assessment at 1, 3, 24, and 36 weeks after surgery. The saline group was taken at 36 weeks. Animals received an overdose of pentobarbitone (100 mg/kg) and were perfused with 50 ml of phosphate buffered saline followed by 250 ml of paraformaldehyde (4% w/v in 0.1M PBS) via transcardiac perfusion. The brains were post-fixed for 2 h in 4% paraformaldehyde and cryo-protected overnight in sucrose (30% w/v in 0.1M PBS) before sectioning on the coronal plane using a freezing microtome (Leica). Sections were collected in 12 series at a thickness of 40 μm. Immunohistochemical detection of NeuN was performed on free-floating sections as previously described in Thompson et al. (2005). Briefly, the sections were incubated overnight with the primary antibody (NeuN, raised in mouse, Millipore) diluted 1:200 in 0.1M PBS with 0.5% Triton X-100 (Amereso, United States) and 5% normal donkey serum (NDS). After washed in PBS the sections were blocked with 2% NDS for 15 min and then incubated for 2 h with biotinylated-donkey-anti-mouse secondary (Jackson labs) diluted 1:400 in PBS with 0.5% TritonX-100 and 2% NDS. The sections were again washed in PBS before incubation with a streptavidin-peroxidase complex (VECTASTAIN ABC system, Vector Labs) for 1 h. Detection of the peroxidase labeled antibody complex was via H_2_0_2_ catalyzed precipitation of the diaminobenzidine (DAB) chromagen. The DAB-labeled sections were dehydrated in alcohol and xylene, and cover-slipped with DePex mounting medium (BDH Chemicals, United Kingdom).

### Quantification of Cortical Volume

A 1:12 series of sections immuno-labeled for NeuN were used to quantify cortical volumes in saline injected animals at 36 weeks and in ET-1 injected animals at 1, 3, 24, and 36 weeks. A Leica (DM6000) microscope equipped with a motorized X–Y stage was used to capture photomontages of whole coronal sections. Cortical area was measured in each hemisphere of every consecutive section beginning 1.7 mm rostral and extending 0.7 mm caudal to Bregma (6 sections). Cortical volumes were calculated from the sum of the area, the section thickness and interval according to the principle of Cavalieri (1966).

### Stereological Quantification of Cortical Neurons

**FIGURE 2:**
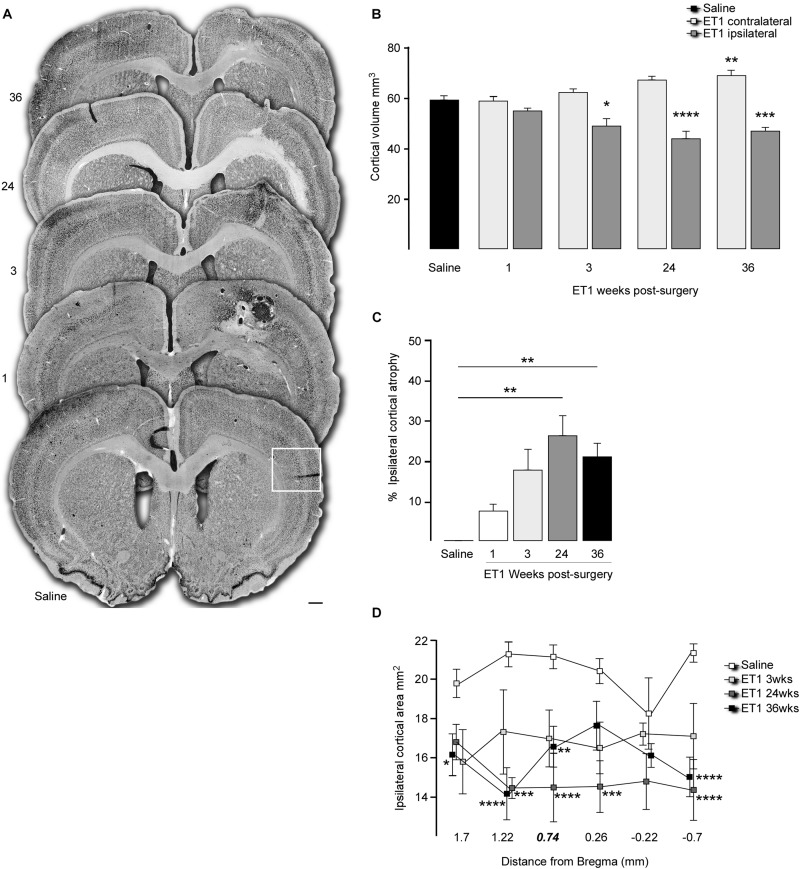
Progressive cortical atrophy after focal ischemia. **(A)** Representative coronal sections labeled for NeuN illustrate gross morphology for a saline injected animal and 1, 3, 24, and 36 weeks after ET-1 injection (note enlarged versions of the boxed area are illustrated in Figure 3). **(B)** Total hemispheric cortical volume (bregma –2.1 to +2.7) in saline injected animals (*n* = 8, pooled data for contralateral and ipsilateral hemispheres shown) and ET-1 animals 1 (*n* = 4), 3 (*n* = 4), 24 (*n* = 5) and 36 (*n* = 8) weeks after ET-1 injection, showing a significant loss of volume in the ipsilateral cortex at 24 and 36 weeks (one way ANOVA, *F*_8_,_41_ = 16.87, *p* < 0.001 with Dunnett’s multiple comparisons tests; ^∗^*p* = 0.0177; ^∗∗^*p* = 0.0046; ^∗∗∗^*p* = 0.0003; ^****^p = 0.0001). **(C)** Progression of ipsilateral cortical atrophy over time, expressed as% atrophy relative to saline treated animals, shows significant atrophy at 24 and 36 weeks (one-way ANOVA *F*_4_,_20_ = 5.65, *p* = 0.0033, Dunnet’s multiple comparison test; ^∗∗^*p* < 0.01). **(D)** Ipsilateral cortical area at specific rostra-caudal levels shows that compared to saline injected animals there was a significant reduction in area initially closest to the ET-1 injection site and then extending to more distal rostro-caudal levels at later time-points [two-way ANOVA with a significance with time *F*_4_,_144_ = 28.83, *p* < 0.0001; saline (*n* = 8), ET-1 3 weeks (*n* = 4), ET-1 24 weeks (*n* = 5), ET-1 36 weeks (*n* = 8); ^∗^*p* < 0.05; ^∗∗^*p* < 0.01; ^∗∗∗^*p* < 0.001; ^****^p < 0.001]. All data shown as the group mean ± SEM. Scale bar: **(A)** 500 μm.

To assess neuronal density in the cortex, the number of NeuN labeled cells were estimated in a defined region of interest immediately adjacent to the infarcted area (lateral and ventral, see boxed area shown in Figure 2A) in order to avoid the vacuous tissue associated with the infarction, which was associated with a high degree of non-specific labeling at the earlier time-points after ET-1 injection. Two sections were used corresponding to approximately 0.78 and 1.74 mm rostral to Bregma. A stereological counting approach with systematic random sampling within the region of interest according to optical dissector rules (Gundersen et al., 1988; Mayhew, 1991) was used to estimate total NeuN cell numbers in that region. Counting frame grid dimensions and fractionator x, y coordinates were determined using the grid overlay program (Stereoinvestigator v7.0, MicroBrightField, Williston, VT, United States). Guard zones were set at 1 μm (top and bottom) and NeuN-labeled nuclei quantified within the counting frame (dimensions used were 40 μm × 40 μm) at periodic intervals (x = 200 μm, y = 200 μm) in the delineated region of interest. Tissue volume was calculated according to the principle of Cavalieri within the Steroinvestigator software in order to determine the density of NeuN cells. The accuracy of the stereological estimations was determined by the coefficients of error and coefficients of variance. Estimations were deemed acceptable if coefficients were > 0.1 (West et al., 1991).

### Statistical Analysis

Unless stated otherwise, all quantitative data is expressed as the mean ± SEM of the mean and an alpha value of < 0.05 was used to define statistical significance. Prism software was used to determine statistical differenced in the means between groups.

Comparison of motor performance between the control and ET-1 treated groups was performed by two-way ANOVA and the Holm-Sidak method was used to correct for multiple comparisons. For comparison of atrophy between the sham group and ET-1 group at multiple time-points and at each Bregma point, one-way ANOVA with Dunnett’s test for multiple comparisons and two-way ANOVA were performed. Only differences between treatment groups at each individual Bregma point were assessed.

## Results

### Motor Function

Animals were tested for gross motor co-ordination using an accelerating rotarod. One week after surgery the ET-1 treated animals showed a significant impairment in their capacity to remain on the rotarod (Figure 1A and Table 1). This resolved by 4 weeks such that the rotarod performance was not significantly different from the saline treated animals for the remainder of the study (Table 1).

**TABLE 1:** Statistical details for behavior tests.

**Test**	**Time points, weeks**	**Saline Mean ± SEM**	**ET-1 Mean± SEM**	
Rotarod	1	295.7 ± 3.28^s^	241.8 ± 12^s^	*t*(11) = 4.025, *p* = 0.002
	4	294.3 ± 1.91^s^	266.5 ± 13.52^s^	*t*(11) = 1.875, *p* = 0.088
	8	300 ± 0^s^	293.4 ± 5.69^s^	*t*(11) = 1.062, *p* = 0.31
	16	300 ± 0^s^	296.5 ± 2.36^s^	*t*(10) = 1.221, p = 0.25
	24	291.2 ± 7.88^s^	286 ± 7.37^s^	*t*(11) = 0.476, *p* = 0.64
	36	287.5 ± 8.12^s^	269.5 ± 21.63^s^	*t*(9) = 0.5995, *p* = 0.56
Staircase % pellets eaten (L/L+R)	2	44.38 ± 1.16	16.94 ± 2.11	*t*(21) = 6.676, *p* < 0.0001
	4	43.22 ± 5.7	18.04 ± 2.24	*t*(21) = 4.874, *p* < 0.0001
	8	43.26 ± 3.7	21.37 ± 2.20	*t*(21) = 4.731, *p* < 0.001
	16	41.72 ± 3.98	22.58 ± 3.1	*t*(21) = 3.019, *p* = 0.0065
	24	42.16 ± 2.87	21.4 ± 1.94,	*t*(21) = 5.182, *p* < 0.0001
	36	41.66 ± 2.93	22.68 ± 1.88	*t*(16) = 5.356, *p* < 0.0001

		**Right side**	**Left side**	

Staircase pellets eaten	2	21.2 ± 1.15	4.7 ± 0.70	*t*(34) = 12.13, *p* < 0.0001
	4	22.1 ± 0.70	5.1 ± 0.70	*t*(34) = 17.07, *p* < 0.0001
	8	24.1 ± 1.07	6.8 ± 0.83	*t*(34) = 12.75, *p* < 0.0001
	16	22.4 ± 1.06	7.1 ± 1.16	*t*(34) = 9.747, *p* < 0.0001
	24	24.9 ± 1.19	7.2 ± 0.84	*t*(34) = 12.18, *p* < 0.0001
	36	23.6 ± 0.75	7.2 ± 0.77	*t*(24) = 15.29, *p* < 0.0001
Adjusted stepping	Forehand left	10.27 ± 0.4	5.671 ± 0.42	*t*(12) = 7.966, *p* < 0.0001
	Forehand right	10.8 ± 0.5944	10.27 ± 0.4799	*t*(12) = 0.697, *p* = 0.4994
	Backhand left	15.2 ± 0.4072	12.49 ± 0.3247	*t*(12) = 5.205, *p* = 0.0002
	Backhand right	15.14 ± 0.1956	14.01 ± 0.4638	*t*(12) = 1.891, *p* = 0.0830

Staircase testing showed that the ET-1 treated animals were significantly impaired in skilled use of the forepaw contralateral to the injected hemisphere relative to the saline treated group (Figures 1B,C). This was evident at the end of the first 10 day testing period (2 weeks after surgery) and was maintained throughout all later testing periods initiated at 4, 8, 16, 24, and 36 weeks. The saline treated animals retrieved similar amounts of pellets with both the right (ipsilateral to injected hemisphere) and left (contralateral) forelimbs at all time-points, while the ET-1 treated animals only retrieved ∼20% of the total pellets retrieved using the contralateral forelimb (Figure 1B) (Table 1). This equated to on average ∼4–5 pellets compared to > 20 pellets retrieved using the ipsilateral forelimb across all time-points (Figure 1C and Table 1). There was no significant difference in the average number of ipsilateral and contralateral pellet retrievals in the saline injected group at any time-point (not shown).

At the final 36-week time-point for motor testing, we elected to include adjusted stepping as an additional test of forelimb use. Compared to the saline control group, animals treated with ET-1 were significantly impaired in their ability to adjust the placement of a weight-baring limb in order to maintain balance as the limb was moved laterally across a smooth surface in the backhand or forehand direction (Figure 1D and Table 1).

### Changes in Brain Volume

Histological analysis 3 days after ET-1 delivery allowed us to calculate the size of the initial cortical infarction, defined as complete absence of NeuN + neurons, as (9.0 ± 2.7 mm^3^; *n* = 4). The remaining ET-1 treated animals were taken for histological assessment at 1, 3, 24, and 36 weeks after injection in order to quantify changes in cortical volume over time. Representative coronal sections immuno-labeled for NeuN at each time-point illustrate the cyto-architectural changes over time (Figure 2A). At the early 1-week time-point an infarcted area around the injected cortical site was clearly apparent, including vacuous tissue architecture characterized by NeuN + cell loss with areas resembling necrosis and edema. The NeuN cell loss extended into the dorsal aspect of the underlying striatum. By 3 weeks the overtly necrotic, infarcted area had largely resolved in most animals and there was gross morphological evidence for glial scarring around the injection site. This persisted at the later 24 and 36-week time-points where there were persistent areas of scarring, including NeuN + cell loss in deep cortical layers proximal to the corpus callosum.

Quantification of total cortical volume between +1.7 to −0.7 mm relative to bregma showed no changed in the ischemic hemisphere relative to saline controls at the early 1 week time-point, but a significant level of global atrophy from 3 weeks that progressed further to 24 and 36 weeks (Figure 2B). Cortical volume analyzed with one way ANOVA, *F*_8_,_41_ = 16.87, *p* < 0.001 and Dunnett’s multiple comparison test: Saline mean ± SEM = 58.98 ± 1.67; 3 weeks ET1 ipsilateral mean ± SEM = 48.67 ± 3.05, *p* = 0.0177; 24 weeks ET1 ipsilateral mean ± SEM = 43.63 ± 2.95, *p* = 0.0001; 36 weeks ET1 ipsilateral mean ± SEM = 46.72 ± 2.03, *p* = 0.0003. Interestingly, there was a progressive increase in contralateral cortical volume, which reached significance compared to saline controls by 36 weeks (36 weeks ET1 contralateral mean ± SEM = 68.7 ± 2.49, *p* = 0.0046) (Figure 2B).

Representation of cortical volume in the ipsilateral hemisphere as a percentage of saline injected animals highlighted a volumetric loss of 26.03 ± 4.99% (*p* = 0.0021) and 20.8 ± 3.44% (*p* = 0.0069) hemispheric volume at the 24 and 36 time-points, respectively (Figure 2C). Inspection of cortical area at specific coronal levels revealed a significant loss in area at later time-points that extended well-beyond the initial injury site to include the most rostral and caudal sections examined (Figure 2D); two way ANOVA, time factor: *F*_4_,_144_ = 28.83, *p* < 0.0001 and multiple comparison at each section distal to Bregma: at 1.7 mm: Saline mean ± SEM: 19.91 ± 0.6, 36 week ET-1 mean ± SEM = 16.28 ± 0.95, *p* = 0.0342; at 1.22 mm: Saline mean ± SEM: 21.39 ± 0.51, 24 weeks ET-1 mean ± SEM = 15.36 ± 0.41, *p* = 0.0004; 36 weeks ET-1 mean ± SEM = 15.23 ± 1.21, *p* < 0.0001; at 0.74 mm: Saline mean ± SEM: 21.27 ± 0.48, 24 weeks ET-1 mean ± SEM = 14.56 ± 1.64, *p* < 0.0001, 36 weeks ET-1 mean ± SEM = 16.68 ± 0.92, *p* = 0.0032; at 0.26 mm: Saline mean ± SEM: 20.53 ± 0.52, 24 weeks ET-1 mean ± SEM = 14.65 ± 1.58, *p* = 0.0006; and at −0.7 mm: Saline mean ± SEM: 21.46 ± 0.34, *p* = 0.0490, 24 weeks ET-1 mean ± SEM = 14.49 ± 1.43, *p* < 0.0001, 36 weeks ET-1 mean 36 week ± SEM = 15.15 ± 0.89, *p* < 0.0001).

**FIGURE 3:**
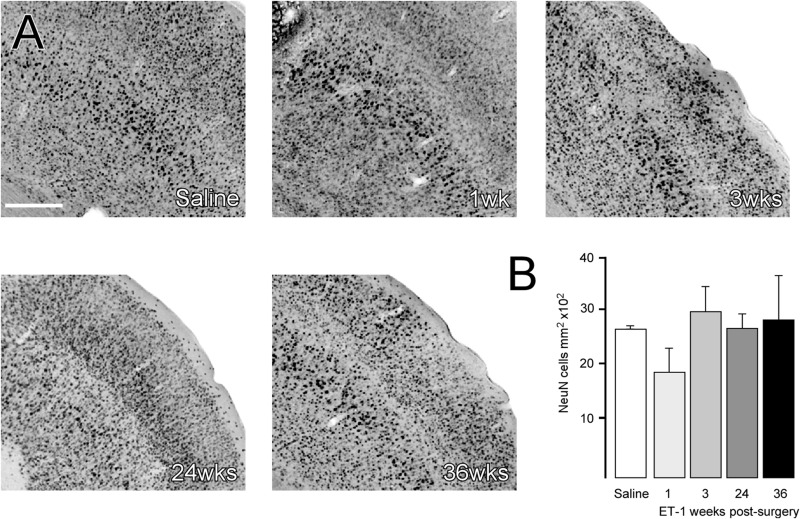
Cortical neuronal cell density does not change in areas of atrophy. **(A)** Representative images of immune-labeling for NeuN in a cortical area adjacent to the injection of saline or 1, 3, 24, or 36 weeks after ET-1 injection. **(B)** Counting of NeuN + cells within a defined area showed that cortical neuronal density was not significantly different in atrophied cortical regions after ischemia [one-way ANOVA *F*_4_,_19_ = 3.059, *p* = 0.042, Dunnett’s *post hoc*; saline (*n* = 6), ET-1 1 week (*n* = 4), ET-1 3 weeks (*n* = 4), ET-1 24 weeks (*n* = 5), ET-1 36 weeks (*n* = 5)]. Scale bar: **(A)** 500 μm.

To determine if changes in brain volume were associated with neuronal loss, stereological counting of NeuN-labeled cortical neurons was performed to determine neuronal density. The area quantified is indicated as a boxed region in the coronal section from the saline group in Figure 2A and representative higher magnification images from each group are shown in Figure 3A. The results showed that neuronal density in the cortical area immediately adjacent to the infarcted area was not significantly different from saline treated animals at any time-point, despite what appeared to be a small reduction at the 1-week time-point (Figure 3B) [one way ANOVA *F*_4_,_19_ = 3.059, *p* = 0.042. Dunnett’s multiple comparisons tests: NeuN density: Saline mean ± SEM = 2687 ±, *n* = 6; ET-1 1 week mean ± SEM = 1896 ±, *n* = 4; ET-1 3 weeks mean ± SEM = 2995 ±, *n* = 4; ET-1 24 weeks mean ± SEM = 2688 ±, *n* = 5; ET-1 36 weeks mean ± SEM = 2852 ±, n-5; *p*-value (saline – ET-1 1 week) = 0.0708; *p*-value (saline – ET-1 3 week) = 0,7365; *p*-value (saline – ET-1 24 week) = 0.999; *p*-value (saline – ET-1 36 week) = 0.9486].

## Discussion

These results show that focal ischemic damage to the frontal cortex through injection of ET-1 can result in impairment of forelimb function that persists for up to 9 months. Gross motor co-ordination, assessed on the accelerating rotarod, was significantly impaired in the acute phase, 1 week after ischemia, but recovered to be similar to control levels at subsequent testing beyond 4 weeks. This is consistent with other pre-clinical studies in various rodent stroke models reporting rapid and sometimes complete recovery in rotarod performance between 4 and 14 days after ischemia (Hunter et al., 2000; Zhang et al., 2000; Bouet et al., 2007). In the first 3 months following human stroke, significant recovery of gross motor function is observed, but patients often report sustained deficits in fine motor function, especially in the upper limb (Jorgensen et al., 1995; Rothrock et al., 1995; Kreisel et al., 2007).

Tests of skilled motor function, particularly paw reaching tasks including the staircase test, have been shown to be more sensitive for detection of motor impairments that persist beyond the spontaneous recovery phase in both mouse (Bouet et al., 2007; Balkaya et al., 2013; Roome et al., 2014) and rat (Gilmour et al., 2004; Windle et al., 2006; Soleman et al., 2010; Trueman et al., 2016) models. The study by Gilmour et al. (2004) has the longest follow up period post-stroke, reporting persistent impairment in a forelimb-reaching task 12 weeks after injection of ET-1 into the sensorimotor cortex. Here we report that impairments of forelimb use in both the staircase and lateralized stepping tests are present up to 9 months after focal cortical ischemia. This is significant for the development of cell replacement based therapies, where therapeutic impact may manifest after months rather than weeks (for review see, Thompson and Bjorklund, 2015). Importantly also in this context, these results were obtained in athymic rats, which allow for the long-term survival of xeno-grafted human cells without immune-rejection.

A conspicuous feature of the histological analysis was the chronically progressive nature of cortical atrophy. The cortical atrophy was extensive and remote from brain infarct site, progressively extending throughout the ipsilesional hemisphere and continuing to progress between the 3 week and 6-month time-points. Counting of NeuN + cell density in the peri-infarct area revealed an initial drop in density at 1 week, while density at the later time-points was similar to saline injected cortex. This suggests *neuronal cell loss* as an underlying feature of the progressive atrophy, rather than a more passive re-organization and shrinkage of the extra-cellular compartment, which would necessarily result in increased neuronal density.

Diffuse and progressive atrophy beyond the penumbra region is increasingly becoming recognized as an important feature of stroke pathophysiology. This is particularly evident from recent clinical imaging studies. Seghier et al. (2014) report progression of ‘whole brain’ atrophy that persists for years after ischemia, and is accelerated in the ipsilesional hemisphere based on T1-weighted imaging of 56 patients 2 months to 6 years after stroke. Other studies suggest patterns of connectivity between remote nuclei and the primary site of damage may well be a major determinant of seemingly diffuse and chronic secondary degeneration. For example, thinning of remote cortical areas has been linked to secondary degeneration within associated white matter tracts of connectivity with the site of infarction (Cheng et al., 2015; Duering et al., 2015). This was also reported in a recent study in rats where ET-1 induced focal ischemia in prefrontal cortex resulted in a pattern of secondary inflammation and white matter damage that matched well with anatomical connectivity to the infarcted area (Weishaupt et al., 2016). In patients, contralesional thalamic volume has also been shown as significantly reduced, with the degree of atrophy proportional to the severity of stroke (Brodtmann et al., 2012; Yassi et al., 2015). The relationship between the development of dementia after stroke and progression of atrophy in brain regions remote to the site of damage is becoming an increasingly important area (Sun et al., 2014). Interestingly, the increased contralateral cortical volume seen here has also been reported in clinical imaging studies (Brodtmann et al., 2012) and may represent a homeostatic, compensatory response.

In summary, we report here that a focal model of cortical ischemia in athymic rats recapitulates important aspects of human stroke relevant for the development of therapies. The stable nature of motor deficit over 9 months on an athymic background forms a valuable model for the assessment of human cell based therapies, where therapeutic impact related to functional integration of mature neurons will likely take months, rather than weeks. Furthermore, the progressive cortical atrophy is in line with findings from recent clinical imaging studies and may be important substrates for stroke-related dementia. Despite the gathering clinical data, this phenomenon has not been well-captured and described in animal models. The utilization of focal ischemic injury models will allow for a systematic approach to developing a better understanding of the relationship between primary sites of injury, the pattern of subsequent secondary degeneration and functional impact, and opportunities for therapeutic intervention, including both protective and regenerative cell-based therapies.

## Data Availability Statement

All datasets generated for this study are included in the article/supplementary material.

## Ethics Statement

The animal study was reviewed and approved by the Florey Institute of Neuroscience and Mental Health Animal Ethics Committee.

## Author Contributions

CE, FS, and T-YW contributed to the experimental procedures and analysis. BK performed the statistical analysis. CP and LT contributed to the conception and design of the study. LT and CE wrote the manuscript. All authors contributed to the manuscript revision, and read and approved the submitted version.

## Conflict of Interest

The authors declare that the research was conducted in the absence of any commercial or financial relationships that could be construed as a potential conflict of interest.
